# New trends of HCV infection in China revealed by genetic analysis of viral sequences determined from first-time volunteer blood donors

**DOI:** 10.1111/j.1365-2893.2010.01280.x

**Published:** 2011-01

**Authors:** Y Fu, Y Wang, W Xia, O G Pybus, W Qin, L Lu, K Nelson

**Affiliations:** 1 Guangzhou Blood CenterGuangzhou, Guangdong, China; 2 The Laboratory of Integrated Biosciences, School of Life Sciences, Sun Yat-sen UniversityGuangzhou, Guangdong, China; 3 Department of Zoology, University of OxfordOxford, UK; 4 Clinical Pathogen Research Center, Third Affiliated Hospital, Sun Yat-sen UniversityGuangzhou, Guangdong, China; 5 Division of Gastroenterology-Hepatology, and Nutrition, Department of Medicine, University of UtahSalt Lake City, UT; 6 Department of Epidemiology, Bloomberg School of Public Health, Johns Hopkins UniversityBaltimore, MD, USA

**Keywords:** blood donor, China, genotyping, HCV, sequence

## Abstract

Recently, we studied hepatitis C virus (HCV) sera-prevalence among 559 890 first-time volunteer blood donors in China. From randomly selected 450 anti-HCV positive donors, we detected HCV RNA in 270 donors. In this study, we amplified HCV E1 and/or NS5B sequences from 236 of these donors followed by DNA sequencing and phylogenetic analysis. The results indicate new trends of HCV infection in China. The HCV genotype distribution differed according to the donors’ region of origin. Among donors from Guangdong province, we detected subtypes 6a, 1b, 3a, 3b, 2a, and 1a at frequencies of 49.7%, 31.0%, 7.6%, 5.5%, 4.1%, and 2.1%, respectively. Among donors from outside Guangdong, we detected 1b, 2a, 6a, 3b, 3a, 6e, and 6n at frequencies 57.1%, 13.2%, 11.0%, 9.9%, 4.4%, 2.2%, and 2.2%, respectively. Although we found no significant differences among regions in age or gender, subtype 6a was more common (*P*< 0.001) in donors from Guangdong than those from elsewhere, whilst subtypes 1b (*P*< 0.02) and 2a (*P* < 0.001) were more frequent outside Guangdong. Disregarding origins, the male/female ratio was higher for subtype 6a-infected donors (*P* < 0.05) than for subtype 1b donors, whilst the mean age of subtype 2a donors was 8–10 years older (*P* < 0.05) than that for all other subtypes. Detailed phylogenetic analysis of our sequence data provides further insight into the transmission of HCV within China, and between China and other countries. The predominance of HCV 6a among blood donors in Guangdong is striking and mandates studies into risk factors for its acquisition.

## Introduction

Hepatitis C virus (HCV) is a blood-borne pathogen that presents a major threat to global public health. Worldwide, about 170 million people are infected with HCV [1], and the prevalence varies among countries [1–6]. HCV can cause chronic liver disease in 75–85% of the infected individuals. The outcomes include liver cirrhosis and hepatocellular carcinoma [7,8]. The rapid, global spread of HCV resulted mainly from transmission through blood transfusion [9]. Recently, in countries where donor screening is performed, new cases are often associated with injection drug use (IDU) and unsafe medical procedures. Other routes are also indicated [10].

Analysis of viral sequences has resulted in classifying HCV into six genotypes and >80 subtypes [11]. HCV genotypes vary in patterns of geographical distribution and therapeutic response. Subtypes 1a, 1b, 2a, 2b, and 3a are ‘global epidemic’. Other genotypes are restricted to particular regions [12–14]. However, the geographical and genetic diversity of HCV is constantly evolving as result of modern transmission and increasing global travel.

Hepatitis C virus classification is usually consistent throughout the genome. Recombination appears rare. Hence, the provisional assignment of HCV genotypes/subtypes can be based on partial genomic regions. Sequences from 5′UTR are optimal for sensitive diagnosis but not sufficient to differentiate subtypes [15,16]. Sequences from the E1 and NS5B regions vary among strains and are suitable for genotyping [12]. Using sequence data from the two regions, we have determined HCV genotype distribution in different areas of China. Overall, 1b is the most predominant followed by 2a. However, in Guangdong Province, 6a has replaced 2a as the second most common subtype [17]. The emergence of 6a is probably because of its close association with IDU. This transmission may have been aggravated in recent years. In our previous report, however, samples were only obtained from patients who may not adequately reflect the general population [17]. In this study, specimens were collected from first-time volunteer blood donors [18]. We performed detailed phylogenetic analysis of E1 and NS5B sequences to provide insights into HCV transmission within China, and between China and other countries.

## Materials and methods

From January 2004 to December 2007, a total of 559 890 first-time volunteer blood donors were recruited. Routine screening detected anti-HCV among 1877 donors. Among the 1877 donors, 450 were selected for RT-PCR, and 270 were found to be HCV RNA+ [18]. cDNA from the latter 270 donors were retained and used in this study.

**Fig. 1:**
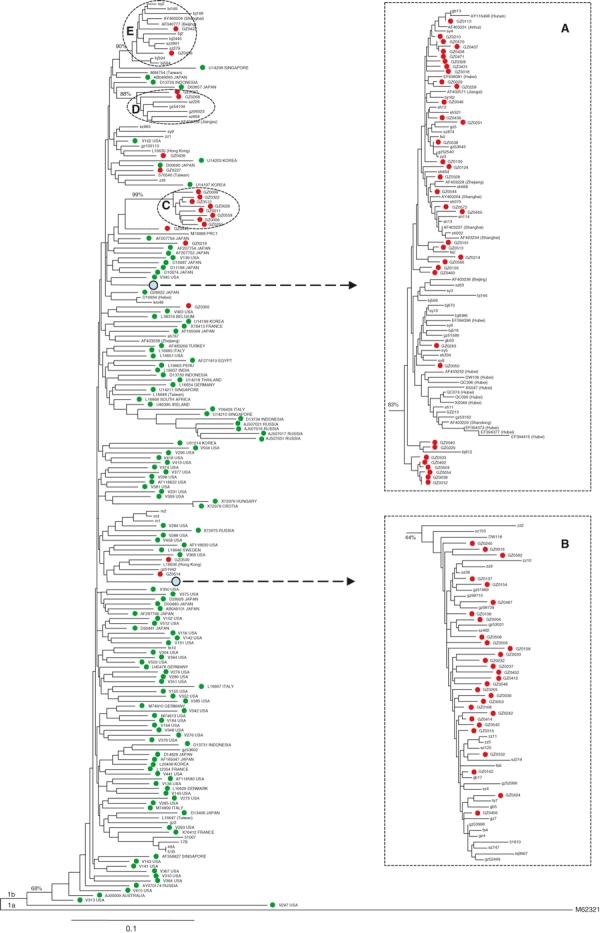
Subtype 1b phylogeny estimated from E1 region sequences (H77 positions: 869–1289). Subtype 1a sequence M62321 was used as an outgroup. Green circles label reference sequences from outside China, and red circles label sequences from this study. Sequences without a circle are Chinese isolates reported in other studies. Blue circles and dashed arrows represent the locations of clusters A and B, which are shown expanded in two boxes on the right. Three dashed circles indicate clusters C, D, and E and bootstrap support values are shown in italics. Scale bar represents 0.10 nucleotide substitutions per site.

**Fig. 2:**
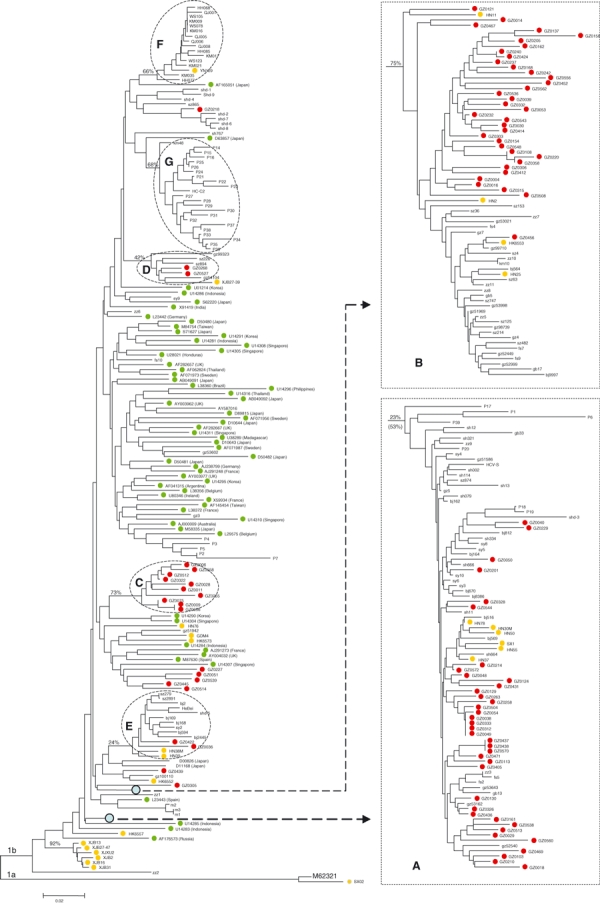
Subtype 1b phylogeny estimated from NS5B region sequences (H77 positions: 8276–8615). Subtype 1a sequence M62321 was used as an outgroup. All labels are the same as those described in the Fig. 1 legend. In addition, yellow circles represent unpublished sequences obtained by us from Chinese patients coinfected with HIV-1 [40], and dashed circles indicate clusters F and G.

**Fig. 3:**
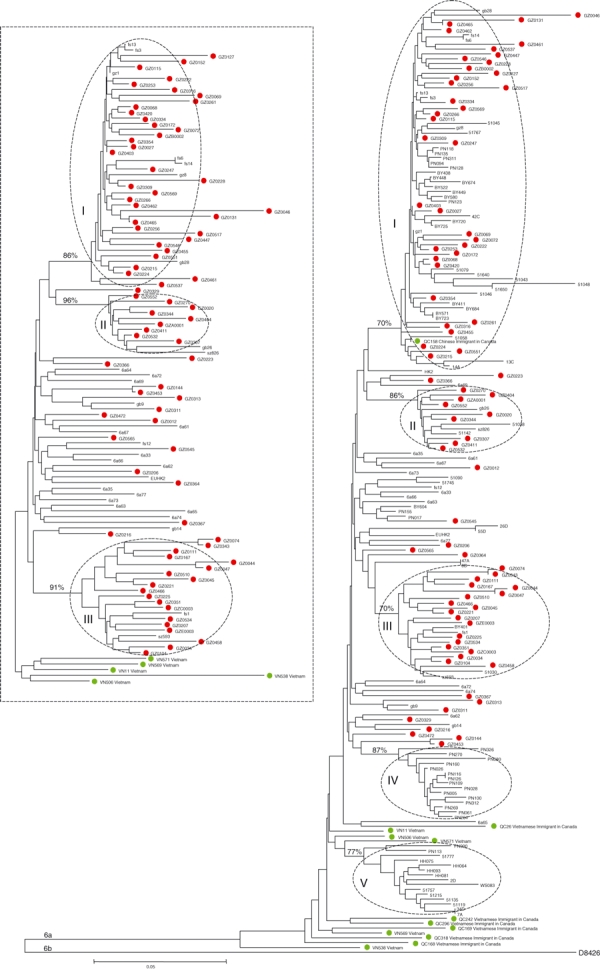
Subtype 6a phylogeny estimated from E1 region sequences (H77 positions: 869–1289). Subtype 6b sequence D84263 was used as an outgroup. All labels are the same as those described in the Fig. 1 legend. In addition, five dashed circles indicate clusters I, II, III, IV, and V. The box on the left contains a smaller phylogeny estimated from longer 510 nt sequences, which shows stronger bootstrap support for clusters I, II, and III.

**Fig. 4:**
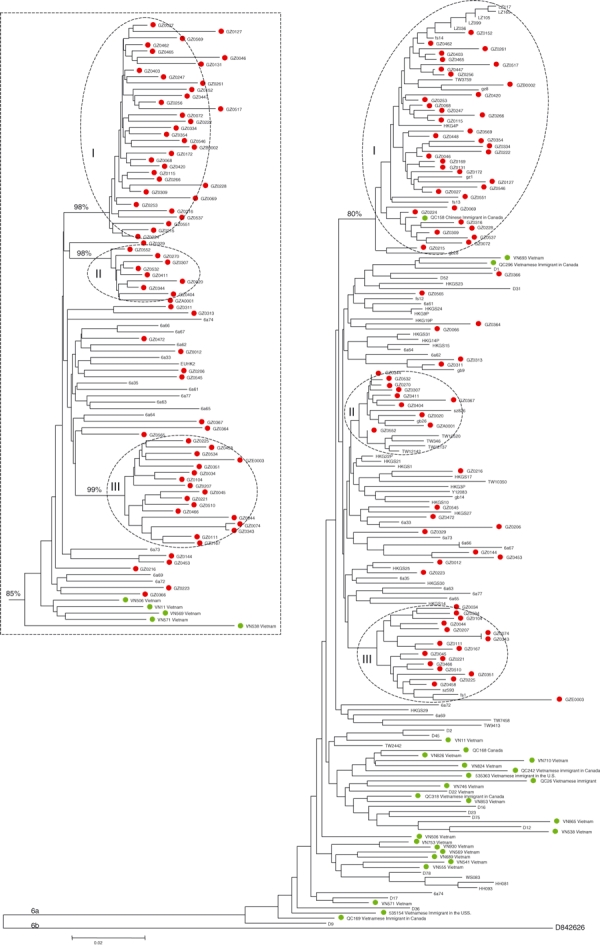
Subtype 6a phylogeny estimated from E1 region sequences (H77 positions: 8276–8615) Subtype 6b sequence D84263 was used as an outgroup. All labels are the same as those described in the Fig. 1 legend. In addition, three dashed circles indicate clusters I, II, and III. The box on the left contains a smaller phylogeny estimated from a longer alignment of concatenated E1 + NS5B sequences (870 nt), which shows very high bootstrap support for clusters I, II, and III.

Sequencing methods were as previously described [17]. Briefly, HCV fragments were amplified using the Primer STAR kit (Takara, Dalian, China). Amplicons were purified with the QIAquick PCR purification kit (QIAgen, Valencia, CA, USA). DNA was sequenced in both directions on an ABI Prism 3100 genetic analyzer (PE Applied Biosystems, Foster City, CA, USA). Sequences were aligned using the CLUSTAL_X program (http://www.geneious.com). Phylogenies were estimated using the maximum-likelihood method under the HKY+I+Γ_6_ substitution model, implemented in the PHYML program (http://atgc.lirmm.fr/phyml/). Bootstrap resampling was performed using 500 replicates. A variety of referenced sequences were retrieved from Genbank and included for analyses (see Figs 1–4).

Guidelines set by the Institution Review Board of the Guangzhou Blood Center and the University of Utah were strictly followed. Written informed consent was obtained from all participants when they donated blood.

## Results

### Characteristics of the study group

Of the 270 donors, 204 (75.6%) were men, and 66 (24.4%) were women; 178 (65.9%) were from Guangdong, and the remainder from elsewhere. Ages ranged from 21 to 54 years with a mean age of 34.4 years (SD = 6.79 years). Two hundred and sixty-eight donors are of Han ethnicity; one each originates from the Zhuang and Tujia ethnic groups [18].

### Hepatitis C virus sequence amplification and classification

Of the 270 donors, E1 and NS5B sequences were amplified from 212 and 229 donors, respectively. E1 and NS5B were both obtained for 205 donors, E1 only for 7 and NS5B only for 24. Collectively, amplification of either or both regions was successful for 236 donors (female/male = 57/179, age = 34.5 ± 6.67 years) but failed in 34 (female/male = 9/25, age = 33.88 ± 6.95). HCV genotypes were determined with the following results: 1b in 97, 6a in 82, 2a in 18, 3a in 15, 3b in 17, 1a in 3, 6e in 2, and 6n in 2 (Table 1).

**Table 1:** Hepatitis C virus genotypes and donor groupings

Genotype	1a (*n* = 3)	1b (*n* = 97)	2a (*n* = 18)	3a (*n* = 15)	3b (*n* = 17)	6a (*n* = 82)	6e (*n* = 2)	6n (*n* = 2)	Total (*n* = 236)
Men*	2	65	13	13	16	67	2	1	179
Women	1	32	5	2	1	15	0	1	57
Age (years)†	31.33 ± 3.11	33.93 ± 7.08	40.83 ± 8.06	33.93 ± 6.75	33.88 ± 6.80	34.34 ± 5.67	30.00 ± 2.00	26.00 ± 3.00	34.46 ± 6.77
E1 + NS5B	3	84	16	9	15	75	1	2	205
E1 only		1	1	1		4			7
NS5B only		12	1	5	2	3	1		24
Guangdong (Group 1)	3	45	6	11	8	72			145
Other areas (Group 2)		52	12	4	9	10	2	2	91

	Total (*n* = 236)		1b (*n* = 97)		6a (*n* = 82)	
Group	Guangdong (*n* = 145)	Non-Guangdong (*n* = 91)	Guangdong (*n* = 45)	Non-Guangdong (*n* = 52)	Guangdong (*n* = 72)	Non-Guangdong (*n* = 10)
Men	116	63	33	32	60	7
Women	29	28	12	20	12	3
Ages (years)	34.40 ± 6.91	34.60 ± 6.56	33.84 ± 7.89	34.00 ± 6.39	34.15 ± 5.80	35.7 ± 4.64

* *P*< 0.05 was observed in 1b comparing with 6a.

† *P*< 0.05 was observed in 2a comparing with 1b, 3a, 3b, and 6a, respectively.

### Analysis of subtype 1b sequences

In total, 97 subtype 1b isolates were identified: 84 were represented by both E1 and NS5B sequences, 1 represented by E1 only, and 12 by NS5B only. Almost all of the E1 sequences were grouped into five clusters, labelled A, B, C, D, and E containing 37, 29, 8, 2, and 2 sequences, respectively. The bootstrap supports for clusters A–E were 83%, 44%, 99%, 88%, and 90%, respectively (Fig. 1). Although many reference sequences were included in the clusters, they were all of Chinese origin [14,17,19–22]. Figure 1 provides an expanded view of cluster A and B (each is collapsed into a branch in the main tree) that were investigated previously and found to coincide with the ‘Cultural Revolution’ in China [23]. These two lineages appear no different to other branches but selectively spread in China. Clusters C, D, and E may have similar epidemiologic histories, although fewer isolates were identified.

The NS5B sequences were also grouped into clusters A, B, C, D, and E, containing 38, 36, 10, 2, and 2 sequences, respectively. Bootstrap supports for clusters A–E were 53%, 75%, 73%, 42%, and 24%. Figure 2 shows two further clusters, F and G, having bootstrap scores of 66% and 68%, respectively. All isolates in cluster F were from Yunnan [24], and all isolates in cluster G were from Beijing [25].

Figures 1 and 2 are compared, and the isolates from this study are located in similar positions, indicating reliable sequencing results and no recent viral recombination events or mixed HCV infection. In total, 87.6% (85/97) of the 1b isolates grouped into clusters A, B, or C (Table 2). Consistent with our previous report [17], cluster A is prevalent nationwide, and cluster B is more common in Guangdong. When the E1 and N5B sequences from identical isolates were concatenated, significant bootstrap supports were obtained for both clusters (not shown).

**Table 2:** Cluster and origin of 1b isolates

Province	A (*n* = 39)	B (*n* = 36)	C (*n* = 10)	D (*n* = 2)	E (*n* = 2)
Chongqing	1				
Guangdong	12	22	6		1
Guangxi	1		2		
Hebei		1			
Henan	3	1			
Hubei	8	1			
Hunan	8	8			
Jiangsu	1				
Jiangxi	2				
Jilin		1			
Liaoning					1
Shandong	1				
Shanxi	1	1	1	2	
Shanghai			1		
Sichuan	1	1			

### Analysis of subtype 6a sequences

Subtype 6a sequences were isolated from 82 donors; E1 and NS5B were sequenced from 75 donors, E1 from 4, and NS5B from 3. The E1 tree presents three clusters (denoted I, II, and III) containing 36, 9, and 19 isolates, respectively (Fig. 3). When 573 nt long sequences were analysed, the three clusters exhibited bootstrap scores of 75%, 91%, and 78%. When sequences were trimmed to 410 nt, bootstrap scores were correspondingly reduced to 70%, 83%, and 45%. The shorter alignment enabled the inclusion of more reference strains, which released two further clusters (denoted IV and V) having bootstrap scores of 90% and 78%, respectively. Clusters IV and V contained isolates from Yunnan, Hubei, and Guangxi [24,26,27]. Sixteen reference sequences were from Hong Kong, but none grouped within clusters I to V. Isolates from IDUs in Guangxi and Hubei appeared throughout the tree [26,27]. Generally, more derived sequences were from China [17], whereas more ancestral sequences were from Vietnam or immigrants from Vietnam.

Among the NS5B sequences, 36, 10, and 16 grouped within clusters I, II, and III, respectively (Fig. 4). Only cluster I had a significant bootstrap score of 80%. In both trees, identical isolates were placed in the same clusters (Figs 3 & 4). Reference sequences of Vietnamese origin tended to locate nearer the tree base. Phylogenetic signal was increased by concatenating E1 and NS5B sequences from identical isolates: the rectangle in Fig. 4 shows the phylogeny. Clusters I, II, and III were again present and supported with bootstrap scores of 86%, 95%, and 88%, respectively. Only five Vietnamese reference sequences could be concatenated; they were placed near the tree root.

### Analysis of subtype 2a sequences

Subtype 2a sequences were isolated from 18 donors; E1 and NS5B were sequenced from 16 donors, whilst E1 only and NS5B only from one each. In both E1 and NS5B trees, subtype 2a isolates do not exhibit a clear geographical pattern (Figure S1). In the E1 tree, we observed three clusters mainly composed of Chinese isolates [17]. The NS5B tree showed two notable clusters: one containing isolates from France and the other representing isolates from a single hospital in Beijing [25].

### Analysis of subtype 3a sequences

Subtype 3a sequences were isolated from 15 donors; E1 and NS5B were from nine donors, E1 only from one, and NS5B only from five. In the both trees (Figures S2 & S3), our majority isolates formed a single cluster, supported by bootstrap scores of 81% and 60%. In the E1 tree, three well-supported UK clusters were observed. In the NS5B tree, there was substantial geographical mixing: isolates from different continents tend to group together, whilst sequences from the same country were spread throughout the tree. Some geographical clusters were apparent but exhibited no strong bootstrap support. In contrast, sequences from China formed two distinct groups.

### Analysis of subtype 3b sequences

Subtype 3b sequences were isolated from 17 donors; E1 and NS5B were from 15 donors and NS5B only from two. Some reference isolates from China were mingled with the strains from this study. They formed a single Chinese cluster with bootstrap support of 78% in the E1 tree and 44% in the NS5B tree. Other reference sequences were from South and Southeast Asia and placed nearer the root of the tree (Figure S4). Hence, subtype 3b likely originated from neighbouring countries and is now growing in China, particularly in Yunnan [24] and Guangxi [26] and among IDUs [27].

### Analysis of subtype 1a sequences

Subtype 1a isolates were sampled from three donors with both E1 and NS5B sequences amplified. We have previously sequenced a 1a strain in a patient from Shanghai [17]. Excluding this, no 1a sequences in Genbank were from China. Phylogenetically, the three 1a isolates appeared not to group with that from the Shanghai patient (Figure S5). In the E1 tree, the three isolates formed a cluster but only exhibited a bootstrap score of 44%. In the NS5B tree, the three isolates were dispersed among strains from other countries. Although six 1a strains from Taiwan formed a small cluster [28], they were not related to the isolates reported here. These results suggest that subtype 1a in China is resulted from sporadic importation events.

### Analysis of subtype 6e and 6n sequences

Subtype 6e and 6n sequences were obtained from two donors each. Phylogenetic analysis grouped the two 6e isolates with GX004 and one 6n isolate close to KM42 (Figure S6). In the E1 tree, the Chinese 6e and 6n clusters were supported by bootstrap scores of 100% and 98%, respectively. In the NS5B tree, no strong support was obtained. GZ0355 was from a donor from Guangxi and was grouped with six subtype 6e isolates all from Guangxi. Similarly, GZ0203 was from a donor from Guizhou and closely grouped with six 6n isolates from Yunnan, which is adjacent to Guizhou [17,24].

### Hepatitis C virus genotype distribution

Hepatitis C virus genotypes were determined from 236 donors. We classified these donors into two groups. The Guangdong group contains 145 donors all from Guangdong Province. The non-Guangdong group contains 91 donors all from other locations (Table 1). Within the Guangdong group, subtypes 6a, 1b, 3a, 3b, 2a, and 1a accounted for 49.7%, 31.0%, 7.6%, 5.5%, 4.1%, and 2.1%, respectively. Within the non-Guangdong group, 1b, 2a, 6a, 3b, 3a, 6e, and 6n accounted for 57.1%, 13.2%, 11.0%, 9.9%, 4.4%, 2.2%, and 2.2%. Between groups, HCV genotype patterns differed. Subtype 6a was more frequent (*P* < 0.001) in Guangdong group; 1b (*P* < 0.02) and 2a (*P* < 0.001) more frequent in non-Guangdong group (Table 1). The donors’ age and gender are not statistically different between groups. HCV subtypes showed no significant relationship with the year of sampling (data not shown).

Taking the 236 donors as a whole, 1b, 6a, 2a, 3b, 3a, 1a, 6e, and 6n accounted for 41.1%, 34.8%, 7.6%, 7.2%, 6.4%, 1.3%, 0.8%, and 0.8%, respectively. This subtype pattern resembles that previously reported [17]. Dividing the donors by HCV subtype, we observed two trends (Table 1). First, the male/female ratio among those infected with 6a was higher (*P* < 0.05) than among those infected with 1b. Second, the mean age of subtype 2a donors was older (*P* < 0.05) than that of donors infected with other strains.

## Discussion

In this study, we observed different patterns of HCV genotype distribution among two groups of blood donors. Subtype 6a was predominant in Guangdong group, whilst 1b and 2a predominant in non-Guangdong group. In 2002, we completed a similar study of 139 patients from nine cities in China. We found that 1b, 2a, and 6a accounted for 66.2%, 13.7%, and 10.1% of infections, respectively. Importantly, among patients from Guangdong, subtype 6a has replaced 2a and become the second most common subtype, accounting for 21.2% (14/66). In contrast, no 6a has been detected among patients from other areas [17]. Findings from the current study indicate further spread of 6a infections in China. Among donors from Guangdong, 6a has become the dominant HCV genotype, accounting for 49.7%. Among donors from other areas, subtype 6a was also detected in 10.6%.

Studies have identified 6a prevalence in Hong Kong, Macau, Thailand, and Vietnam. Other studies have found 6a in Singapore (U908306-U908309) and Taiwan [28]. In Hong Kong, 6a has been detected in 27–30% of HCV-infected donors and 60% of HCV-infected IDUs. In Hong Kong, 6a appears to spread to the general population mainly through IDUs. We have postulated that subtype 6a in Guangdong was introduced from Hong Kong [17], because of the geographical proximity of the two locations and because the subtype was detected earlier in Hong Kong. Whilst this may be true for some cases, it is not sufficient to explain the recent 6a spread in mainland China. Phylogenetically, 6a sequences from Guangdong formed three clusters (denoted I, II, and III). Cluster I also contained sequences from IDUs from different regions of China, including cities in Guangxi province bordering Vietnam [26], Liuzhou in Guangxi [29], and Wuhan in Hubei province [27]. Clusters I and II may represent 6a strains originating in Guangdong and now starting to seed IDU networks elsewhere. Five subtype 6a isolates from IDUs in Taiwan [28] also grouped in cluster II, indicating the IDU networks extended from mainland China to Taiwan. Spreading of HIV in IDUs via this route has also been reported [30]. Cluster VI was among IDUs in Guangxi only. Cluster V could be an outcome of interchange between IDU networks in Yunnan and Guangxi [25–27,31]. Phylogenetically, subtype 6a sequences from Hong Kong are distinct from those isolated in mainland China [17], suggesting that the 6a circulation in mainland China may not be directly linked to that in Hong Kong.

The causes of 6a emergence in China are unknown. Based on phylogenies, we hypothesize that subtype 6a originated in Vietnam, or perhaps pre-existed in southern China. A possible historical event for the importation of 6a to China was the emigration of 290 000 Vietnamese to China from the late 1970s to early 1980s [32]. Hong Kong also received 100 000 such emigrants, mostly from South Vietnam [33]. Many ethnic Chinese from North Vietnam fled to China and were resettled in Yunnan, Guangxi, and Guangdong provinces [34].

Some 6a strains may have entered the blood transfusion networks and undergone transmission among recipients, particularly before the discovery of HCV in 1989, or through unsafe medical procedures prior to the governmental ban on paid blood donors in 1998 [35]. Although no large-scale transfusion-linked infections were recorded, some individuals could have been infected by the use of contaminated plasma or unscreened medical infusions, such as foetal liver cells and therapeutic immunocytes that were injected commonly in China during 1990s [36,37].

Guangdong was the first region of mainland China to undergo economic reforms because of its proximity to Hong Kong. It was opened to outside investment and has undergone rapid economic growth. This has resulted in greater social exchange with other countries and has attracted millions of internal ‘migrant labourers’ [38]. The numbers of commercial sexual workers and IDUs have risen alongside this economic growth, providing more opportunity for 6a strains to spread to the general population [39].

In Guangdong, the relative prevalence of 6a has grown from undetectable levels to >20% of infections [17]. In this study, we demonstrate further 6a growth, such that it has become the dominant HCV strain, infecting 49.7% of donors in this study. The spread has occurred despite the outlawing of paid blood donors [35] and improved healthcare standards. Other transmission routes most likely explain the growth of 6a infection. Recent increases in the numbers of IDUs have been reported. IDUs can transmit 6a strains to distant areas, via known drug trafficking routes [31].

This is the first study of HCV genotype distribution among first-time volunteer blood donors in China. Subtype 1b was the most common, accounting for 41.1%. Most of these isolates were classified into two clusters, A and B. This is consistent with our previous study [17] and indicates that the geographical distribution of the two clusters in China remains largely unchanged. Overall, a marked decrease in the proportion of 1b infections was observed, down from 66.2% (92/139) in our previous report [17] to 41.1% (97/236) in the present study. Explanations are the relative increase in 6a infections and/or a larger portion of donors from Guangdong. However, if we consider only donors from outside Guangdong, a decreased 1b frequency is still observed (57.1%). Subtype 1b strains are likely more associated with transmission via blood transfusion and medical procedures. Effective measures to reduce these transmissions have lead to decreased 1b infections. In contrast, subtype 6a strains may be more linked to IDU and sexual transmission, which are both increasing risks in Guangdong.

We found that subtype 2a infected donors were significantly older (8–10 years) than those infected by other HCV strains. Phylogenetically, 2a sequences from China did not form clear geographical clusters. The relative prevalence of 2a has declined in comparison to our previous report [17], which is apparent when the Guangdong group and non-Guangdong group are considered separately or as a whole. Collectively, it is suggested that new cases of HCV 2a infection in China have been reduced.

## Abbreviation:

HCV,: hepatitis C virus

## SUPPORTING INFORMATION

**Figure S1** Subtype 2a phylogeny estimated from (A) E1 region (H77 positions: 869-1289) and (B) NS5B region (H77 positions: 8276-8615). Subtype 2b sequence D10988 was used as an outgroup. Green circles label reference sequences from outside China, red circles label sequences from this study and yellow circles label Chinese isolates reported in other studies.

**Figure S2** Subtype 3a phylogeny estimated from E1 region (H77 positions: 869-1289). Subtype 3b sequence D49374 was used as an outgroup. All labels are the same as those described in the Figure S1.

**Figure S3** Subtype 3a phylogeny estimated from NS5B region (H77 positions: 8276-8615). Subtype 3b sequence D49374 was used as an outgroup. All labels are the same as those described in the Figure S1. The symbol ‘//’ denotes where this large phylogeny has been split into two parts.

**Figure S4** Subtype 3b phylogeny estimated from (A) E1 region (H77 positions: 869-1289) and (B) NS5B region (H77 positions: 8276-8615). Subtype 3a sequence D17763 was used as an outgroup. All labels are the same as those described in the Figure S1.

**Figure S5** Subtype 1a phylogeny estimated from (A) E1 region (H77 positions: 869-1289) and (B) NS5B region (H77 positions: 8276-8615). Subtype 1b sequence M58335 was used as outgroup. All labels are the same as those described in the Figure S1.

**Figure S6** Subtype 6e and 6n phylogeny estimated from (A) E1 region (H77 positions: 869-1289) and (B) NS5B region (H77 positions: 8276-8615). Subtype 2a sequence D00944 was used as an outgroup. All labels are the same as those described in the Figure S1.
