# Supplementary material for: New trends of HCV infection in China revealed by genetic analysis of viral sequences determined from first-time volunteer blood donors
Source: J Viral Hepat. 2011 Jan;18(1):42–52. doi: 10.1111/j.1365-2893.2010.01280.x (PMC3020328; doi:10.1111/j.1365-2893.2010.01280.x)
Supplement: Supplementary file 1 [file jvh0018-0042-SD1.doc]

Supplementary Figure 1

Supplementary Figure 2

Supplementary Figure 3

Supplementary Figure 4

Supplementary Figure 5

Supplementary Figure 6
